# Association Between the Medicare Advantage Quartile Adjustment System and Plan Behavior and Enrollment

**DOI:** 10.1001/jamahealthforum.2023.4822

**Published:** 2024-01-12

**Authors:** Roslyn C. Murray, David J. Meyers, Erin C Fuse Brown, Travis C. Williams, Andrew M. Ryan

**Affiliations:** 1 University of Michigan, Ann Arbor; 2 Brown University, Providence, Rhode Island; 3 Georgia State University, Atlanta

## Abstract

**Importance:**

Medicare Advantage (MA) has grown in popularity, but critics believe that insurers are overpaid, partially due to the quartile adjustment system that determines plan benchmarks. However, elimination of the quartile adjustments may be associated with less generous benefits and fewer plan offerings, which could slow MA enrollment growth.

**Objective:**

To examine whether the quartile adjustment system is associated with differences in county-level benefits, insurer offerings, and MA enrollment.

**Design, Setting, and Participants:**

The quartile adjustments create discontinuous jumps in county-level base payments based on historical traditional Medicare spending. Data from January 2017 to December 2021 and a regression discontinuity design were used to examine changes in insurer behavior and MA enrollment between quartiles. The analytic sample included 1557 county observations.

**Main Outcomes and Measures:**

Study outcomes included monthly premiums, the share of plans charging premiums, primary care copayments, the share of plans using rebates to reduce Part B premiums, supplemental benefits, plan and contract availability, and MA enrollment.

**Results:**

Discontinuities were found in the quartile adjustments and benchmarks. A 1–percentage point (pp) increase in the quartile adjustment was associated with a $6.36 increase in monthly benchmarks (95% CI, 5.10-7.62), a $0.51 decrease in monthly premiums (95% CI, −0.96 to −0.07), and a 0.68 pp decrease in the share of plans charging premiums (95% CI, −1.25 to −0.10). Significant changes were not found in primary care copayments (-$0.04; 95% CI, −0.17 to 0.09), the share of plans using rebates to reduce Part B premiums (−0.17 pp; 95% CI, −0.34 to 0.01), supplemental benefits (eg, preventive dental coverage; 0.17 pp; 95% CI, −0.25 to 0.0), the number of plans (1.06; 95% CI, −3.44 to 5.57) or contracts (0.31; 95% CI, −0.18 to 0.81), or the MA enrollment rate (0.16 pp; 95% CI, −0.61 to 0.94).

**Conclusions and Relevance:**

The study results suggest that MA plans are not very sensitive to modest changes in payment rates. Modifications to the quartile adjustment system may generate savings without substantially affecting MA beneficiaries.

## Key Points

**Question:**

Is the quartile adjustment system associated with differences in county-level benefits, insurer offerings, and Medicare Advantage (MA) enrollment?

**Findings:**

In this cross-sectional study of 1557 county observations, an examination of discontinuous jumps in quartile adjustments that determine MA benchmarks with a regression discontinuity design found limited evidence that the quartile adjustments were associated with changed plan benefits and offerings or enrollment at the quartile cutoffs.

**Meaning:**

The results of this study suggest that modifications to the quartile adjustment system that determines MA benchmarks may reduce extra payments to plans and may not have significant negative associations for beneficiaries.

## Teaser

This cross-sectional study examines the association of the quartile adjustment system with differences in county-level benefits, insurer offerings, and Medicare Advantage enrollment.

## Introduction

Due to attractive benefits, little to no premiums, and low cost-sharing, Medicare Advantage (MA), which allows private insurers to offer health benefits to Medicare beneficiaries, has grown in popularity. More than 50% of Medicare beneficiaries are now enrolled.^1^ The US Centers for Medicare & Medicaid Services (CMS) provides a per beneficiary per month payment to MA plans to manage the care of enrollees. Proponents argue that the program provides strong incentives for efficiency and enhancements to health care quality and beneficiary outcomes.^2,3,4,5,6^ Yet critics believe that insurers are overpaid due to excessive coding of patient risk that inflates payments, quality bonuses, and the quartile adjustment system, which determines plan base payments.^7,8,9^

While risk adjustment and upcoding in MA have been criticized widely, the quartile adjustment system has received somewhat less attention.^10^ Within this system, CMS sets payments to MA plans based on historical per capita spending among traditional Medicare (TM) beneficiaries in a county. Counties are classified into quartiles according to their historical TM spending calculated during the previous year (t-1). The historical TM spending calculated during the current year (t) is adjusted based on quartile classification to determine the base benchmark. For counties in the lowest spending quartile (quartile 1), a 115% adjustment is applied to the historical TM spending, 107.5% for counties in quartile 2, 100% in quartile 3, and 95% in quartile 4. In the year when a county’s quartile designation shifts, it is paid the average between these quartiles during this transition period.^11^ There may be additional adjustments for quality, enrollment, and other factors (see the eMethods in Supplement 1 on benchmark setting). One rationale behind the quartile adjustments was to encourage MA insurers to participate in low-spending counties.^12^ Yet evidence suggests that this has been associated with sizeable overpayments to counties with comparably lower TM spending.^13,14^

The association of the quartile adjustments with plan behavior and program outcomes is not fully understood. As a county crosses a quartile cutoff, it receives lower benchmark payments despite typically having experienced at most modest increases in TM spending. This may be followed by less generous benefits and fewer plan offerings, which could be associated with slower MA enrollment growth. Most research examining plan responses to payment changes was conducted before the Affordable Care Act (ACA) and current quartile adjustment system.^15,16,17,18,19^ Studies examining payment changes since the transition to the ACA payment structure have found limited associations with enrollment and modest reductions in some plan benefits.^20,21^ In this study, we used a novel source of variation to evaluate the association of the quartile adjustments with insurer behavior and enrollment.

## Methods

### Data Sources and Sample Population

We used county-level MA Ratebook data from 2017 to 2021. We used 2017 as the first year of analysis because the phase in to post-ACA benchmarks was complete.^11^ The Ratebook data include historical TM spending, quartile adjustments, and county-level benchmarks. We used the MA Contract and Enrollment files to calculate the number of MA contracts and plans offered per county per year as well as the generosity of the benefits offered by plans in the county. We also used the Geographic Variation Public Use File, which includes county-level MA enrollment rates (ie, the percentage of Medicare beneficiaries enrolled in MA) and beneficiary data, such as age, gender, and number of chronic conditions. Finally, we used Census data to identify urban counties and population size and data from the American Community Survey to identify county-level unemployment rates and per capita income.

For the main analysis, we excluded county-years that were missing covariates and county-years subject to pre-ACA benchmark caps (3907 county-years; unique counties, 1122). In these counties, quartile adjustments are not used to modify payments.

### Outcomes

In response to payment changes, insurers may modify the benefits offered to beneficiaries. The benefits outcomes included (1) monthly premiums, (2) primary care copayments, (3) the share of plans charging premiums, (4) the share of plans using rebate dollars to reduce Part B premiums, and (5) the share of plans offering supplemental benefits (ie, preventive dental coverage, comprehensive dental coverage, coverage for eye examinations, coverage for eyewear, coverage for hearing examinations, and coverage for hearing aids). Benefits measures are reported at the plan level. To identify county-level measures, we generated an average weighted by plan enrollment in the county.

Insurers may also change plan offerings in a county. Therefore, we examine the number of plans and contracts available at the county level. Each insurer may have multiple contracts in a county with similar networks, and each contract may offer multiple plans. Insurers may find it easier to change plan offerings before deciding to eliminate a contract. Finally, we examined changes in the MA enrollment rate, which may be expected to change in response to less generous benefits and fewer plan offerings.

### Statistical Analysis

Standard regression models may not account for unobserved factors that affect insurer behavior and are associated with quartile placement. For example, counties in the highest-spending quartile may have more plans, more generous plans, and greater enrollment because they are in urban areas or due to historic patterns of managed care penetration.^22,23,24^ To address this potential bias, we used discontinuities in payments due to the quartile adjustment system using a regression discontinuity design to evaluate changes in insurer behavior and MA enrollment. If counties just greater and less than the quartile cutoffs were the same, except for the quartile adjustments, then any differences in the outcomes could be attributed to differences in MA payment.

However, not all counties that cross the threshold get the relevant quartile adjustment. Counties that transition quartiles get an adjustment that is the average of the current and prior year adjustment amount. Therefore, we used a fuzzy design, in which the first stage uses the county’s quartile placement to instrument for the quartile adjustment in the second stage. The running variable was the historical TM spending calculated during the previous year, which determined counties’ current year quartile classification and adjustment. We rescaled this measure to represent the distance from the closest cutoff. We used indicator variables for the county quartiles as instruments. We controlled for which of the 3 cutoffs (ie, 25th, 50th, and 75th percentile) the county observation was subject to allow for different intercepts for the association between the running variable and quartile adjustments around these 3 cutoffs. We allowed the fit to vary by cutoff. We estimated models at the county level. Control variables included average beneficiary age, the share of female individuals, unemployment rates, per capita income, average beneficiary risk scores, the share of the population in urban areas, and the county population size (see the eMethods in Supplement 1 for regression specifications).

Plans may be less likely to make adjustments to plan benefits and offerings due to their quartile placement in 1 year. So we classify a county’s quartile based on their median quartile position during the study period (2017-2021). The analysis used the county-level mean of other covariates and outcomes. Finally, we restricted the analysis to counties with prior year historical TM spending within $25 less than and greater than each of the 3 cutoffs to facilitate interpretation and ensure that a county observation was only subject to 1 of the 3 cutoffs. The dollar difference between the 25th and 50th percentile cutoff is approximately $50. The dollar difference between the 50th and 75th percentile cutoff is approximately $65. By limiting the bandwidth to $25, we would avoid double counting a county that is subject to 2 cutoffs. For example, a county in quartile 2 could be above the 25th and below the 50th percentile cutoff. We kept the observation and cutoff that was within the $25 bandwidth. The final sample included 1557 county observations.

In sensitivity analyses, we altered various aspects of the model, including bandwidth ($20, $30, and $35) and functional form. We also examined whether changes in the quartile adjustments were associated with insurer behavior in future years by estimating additional models in which the running variable and instruments were specified as 1-year, 2-year, and 3-year lags. Finally, because benefits are at the plan level and could span multiple counties, we examined a subset of the benefits outcomes for plans whose service area was made up of 1 county.

All analyses were weighted by the county-level population of Medicare beneficiaries. We used robust standard errors. All analyses were performed using Stata, version 17 (StataCorp), and statistical significance was set at *P *< .05.

## Results

Monthly benchmarks were highest for counties near the 75th percentile cutoff. Average monthly premiums and out-of-pocket maximums were highest for counties near the 25th percentile cutoff. The highest share of plans charging premiums were around the 25th percentile cutoff and the highest share of plans using rebate dollars to reduce Part B premiums were around the 75th percentile cutoff. Average primary care copayments and the share of plans offering supplemental benefits were approximately similar across the quartile cutoffs. Counties near the 75th percentile cutoff had more plans and contracts than counties near other cutoffs, but counties closer to the 25th and 50th percentile cutoffs had higher MA enrollment rates (Table).

**Table.** Characteristics of Included Counties by Quartile Cutoff^a^^,^^b^

Characteristic	Mean (SD)		
	25th Percentile cutoff	50th Percentile cutoff	75th Percentile cutoff
No. of counties	660	742	688
Medicare beneficiaries per county	66 392 (66 014)	69 991 (70 319)	193 200 (198 167)
Monthly benchmark, $	890.0 (30.5)	886.7 (27.3)	906.3 (16.4)
Monthly premiums, $	10.8 (8.4)	10.6 (8.1)	10.3 (8.0)
Primary care copayment, $	8.2 (2.3)	8.3 (2.3)	8.3 (2.1)
% of Plans charging >$0 premiums	31.6 (10.5)	30.6 (10.5)	27.7 (9.7)
% of Plans using rebates to reduce Part B premium	6.1 (2.8)	6.4 (3.4)	6.7 (3.3)
% of Plans offering preventive dental	86.6 (7.0)	87.9 (6.3)	84.6 (6.2)
% of Plans offering comprehensive dental	71.3 (9.2)	73.5 (7.7)	71.2 (6.8)
% of Plans offering eye examinations	95.7 (3.6)	96.2 (3.1)	95.2 (3.3)
% of Plans offering eyewear	82.2 (8.7)	83.4 (8.4)	84.1 (6.5)
% of Plans offering hearing examinations	84.5 (7.1)	86.1 (6.3)	86.3 (4.4)
% of Plans offering hearing aids	76.7 (8.4)	78.4 (7.2)	77.6 (5.3)
No. of plans	611.7 (85.6)	622.8 (96.3)	725.7 (161.1)
No. of contracts	119.4 (8.1)	119.8 (7.7)	123 (7.8)
% of Beneficiaries enrolled in MA	45.6 (13.5)	41.2 (13.3)	40.5 (13.9)

Abbreviation: MA, Medicare Advantage.

^a^ Source: Authors’ analysis of 2017-2021 MA Ratebook data.

^b^ Excludes counties that are subject to the pre–Affordable Care Act benchmark cap.

**Figure 1.**
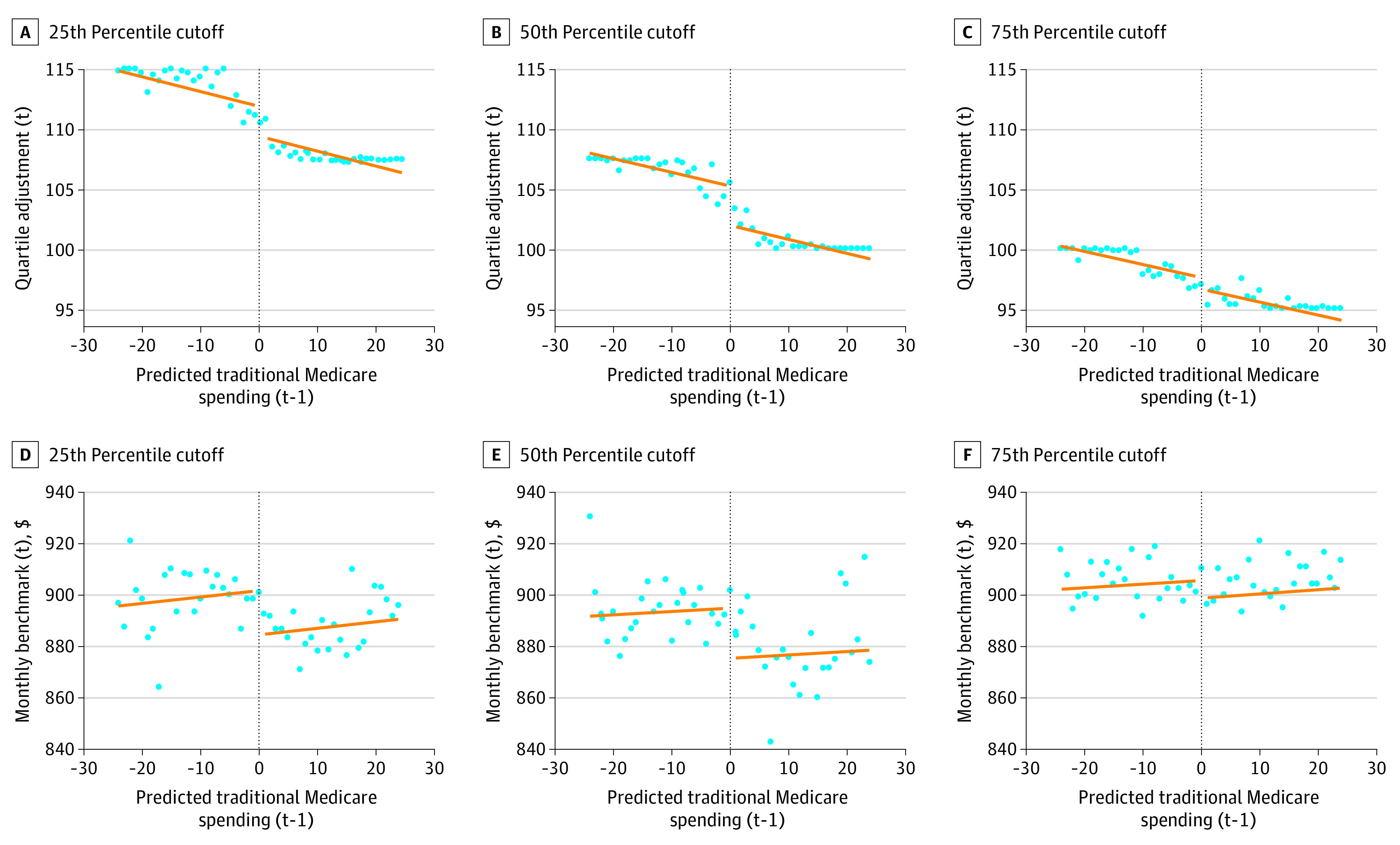
Average Change in Quartile Payment Adjustments and Benchmarks at Quartile Cutoffs Source: authors' analysis of 2017 to 2021 Medicare Advantage Ratebook data. The blue dots represents the average for quartile adjustment (t) or benchmark (t) for counties that have a prior year predicted traditional Medicare (TM) spending (t-1) that are greater than or less than the cutoff. The orange line represents the fitted association between the previous year predicted TM spending (t-1) and the quartile adjustment (t) or benchmark (t). The dotted lines represent the quartile cutoffs (t), for which counties experience different adjustments to their current year predicted TM spending (t) to identify county-level benchmarks. There are discontinuities in the quartile adjustments at the cutoffs that translate into differences in monthly benchmarks at the cutoffs.

The analytic sample included 1557 county observations. The first stage showed expected reductions in the quartile adjustment amount and monthly benchmarks at the cutoffs (Figure 1). The difference in the quartile adjustment for counties just greater than the 25th percentile cutoff compared with those just below was −3.78 percentage points (pp) (95% CI, −4.72 to −2.83), the difference for counties at the 50th percentile cutoff was −4.49 pp (95% CI, −5.20 to −3.78), and the difference for counties at the 75th percentile cutoff was −2.01 pp (95% CI, −2.79 to −1.23). We found that a 1-pp increase in the quartile adjustment was associated with a $6.36 increase in county monthly benchmarks (95% CI, 5.10-7.62). Combining these estimates, the difference in benchmarks at the 25th percentile cutoff was −$24.04 per beneficiary per month (a 2.7% decrease), −$28.56 (a 3.2% decrease) at the 50th, and −$12.78 (a 1.4% decrease) at the 75th percentile cutoff (eFigure 1 in Supplement 1).

**Figure 2.**
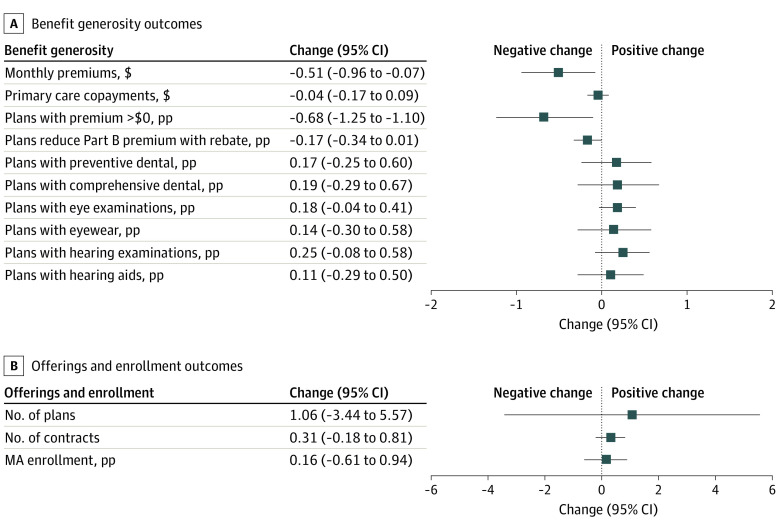
Association of a 1–Percentage Point (PP) Increase in the Quartile Adjustment With Outcomes Source: authors' analysis of 2017 to 2021 Medicare Advantage (MA) Ratebook data. The forest plots show that there were no differential changes in most benefit generosity outcomes and the number of plans, contracts, and MA enrollment. There were significant reductions in premiums and plans charging premiums.

Higher quartile adjustment factors were associated with slightly lower premiums (eFigure 2 in Supplement 1). A 1-pp increase in the quartile adjustment was associated with a $0.51 decline in monthly premiums (95% CI, −0.96 to −0.07) and approximately −$1.93, −$2.29, and −$1.03 per beneficiary per month at the 25th, 50th, and 75th percentile cutoffs, respectively (Figure 2). We similarly found that the share of plans charging premiums greater than $0 declined by 0.68 pp due to a 1-pp increase in the quartile adjustment (95% CI, −1.25 to −0.10) (Figure 2; eFigure 3 in Supplement 1). This translated to a decline in the share of plans charging premiums by 2.57 pp, 3.05 pp, and 1.37 pp at each of the 3 cutoffs. We did not find significant changes in primary care copayments (−$0.04; 95% CI, −0.17 to 0.09), the percentage of plans using rebates to reduce Part B premiums (−0.17 pp; 95% CI, −0.34 to 0.01), and the percentage of plans offering supplemental benefits (eg, preventive dental coverage; 0.17 pp; 95% CI, −0.25 to 0.60) (Figure 2; eFigures 4-6 in Supplement 1). There was limited evidence of changes in insurer offerings at the cutoffs due to a reduction in the quartile adjustment. We found that an increase in the quartile adjustment was associated with a nonsignificant increase of 1.06 additional MA plans (95% CI, −3.44 to 5.57) and 0.31 additional contracts (95% CI, −0.18 to 0.81), as well as a 0.16-pp increase in the MA enrollment rate (95% CI, −0.61 to 0.94) (Figure 2; eFigure 7-9 in Supplement 1).

Results were very similar when we altered the bandwidth and functional form (eFigures 10-13 in Supplement 1). Further, we found no evidence that the association between quartile adjustments and the study outcomes changed when evaluated during a longer time horizon (eFigures 14-16 in Supplement 1). Our findings were also consistent when examining associations in plans with a presence in only 1 county, but they were not statistically significant (eFigure 17 in Supplement 1).

## Discussion

The results of this cross-sectional study suggest that a decline in the quartile adjustment was associated with a decline in plan benchmarks and small increases in monthly premiums and the propensity to charge premiums. However, we found small and nonsignificant changes in primary care copayments, the percentage of plans using rebates to reduce Part B premiums, the percentage of plans offering supplemental benefits, the number of plans and contracts offered by insurers, and MA enrollment rates.

Our findings are consistent with the broader literature examining the association between benchmark changes and benefit generosity, insurer offerings, and enrollment. Most research examining plan responses to payment changes was conducted before the ACA and current quartile adjustment system. This research has generally found limited associations of higher payment with plan offerings and enrollment.^15,16^ But early literature has estimated that approximately 12.5% to 54% of higher payments are passed on to MA beneficiaries via lower premiums or cost-sharing and supplemental coverage.^16,17,18,19^ Our findings complement 2 recent articles examining changes in MA payment after the ACA. A study using variation in plan-level benchmarks from 2012 to 2019 found that some benefits could potentially be lost due to benchmark reductions. However, the associations with premiums, cost-sharing, and supplemental benefits were modest.^20^ Another article, using data from 2008 to 2019, found that there were no significant differences in enrollment in counties that faced large payment cuts due to the ACA compared with counties with small cuts.^21^ Our finding that exogenous changes to plan payments were associated with little to no change in plan offerings, benefits, and enrollment is consistent with these articles. Yet our examination of the quartile payment system, regression discontinuity identification strategy, and use of more data (2020 and 2021) are new contributions to the literature.

There are a few reasons why MA insurers and beneficiaries may not be sensitive to changes in payment due to the quartile adjustments. First, it is widely known that insurers benefit from large payment subsidies through upcoding, quality bonuses, benchmark setting, and favorable selection.^1,25^ These subsidies largely support plan profits, as research has demonstrated that plans pass a small share of payment increases to reduce beneficiary premiums and cost-sharing.^13,16,26^ Therefore, modest reductions in benchmarks through the quartile adjustments may be partially absorbed by plans and not passed along to beneficiaries through less generous benefits and fewer offerings. Second, plans span multiple counties; thus, they may be in multiple quartiles facing different adjustments in a given year. Insurers may not be influenced to make substantial changes to their plans given a specific quartile designation. Finally, enrollment is sticky and likely slow to respond to changes in insurer payment.^27,28^ Beneficiaries prefer to stay in plans they have chosen. Plan switching is largely associated with changes in cost-sharing and benefits.^29^ Because we found that benefits did not change around the cutoffs, it follows that switching would have been limited.

### Policy Implications

Our findings suggest that MA insurers are not sensitive to payment differences due to the quartile adjustments. Therefore, modifications to the quartile adjustment system in line with our estimates may generate savings for the Medicare program without concomitant associations with benefits, offerings, and MA enrollment.^13,14,30^

The quartile adjustment system overpays plans in areas with low TM spending and generates sizable differences in payment across quartile designations. The Medicare Payment Advisory Commission (MedPAC) estimates that plans in low-spending counties are paid 9% more than local TM spending when accounting for quality bonuses.^13,14^ Thus, MedPAC has proposed an alternative payment system that would institute a 50%/50% blend of per capita local and price-standardized national TM spending. This change would move all benchmarks closer to TM spending but keep benchmarks in low-spending areas higher than and benchmarks in high-spending areas lower than TM spending. It would also eliminate arbitrary discontinuities in payment for counties just greater and less than the quartile cutoff. The Congressional Budget Office estimates that this recommendation would save more than $10 billion over 5 years. MedPAC does not expect that it would have adverse associations with MA enrollment, plan offerings, or supplemental benefits.^13^ Our research supports the expectation that modifications to the quartile payment system are not likely to significantly disadvantage MA beneficiaries. However, changes to the quartile adjustment system would require Congressional action.^9^

### Limitations

Our study had several limitations. First, our regression discontinuity analysis relied on the assumption that counties on either side of the quartile cutoffs were the same except for the quartile adjustment that was applied to the county’s historical TM spending. To examine this assumption, we tested whether county-level covariates also differed at the cutoffs, finding no evidence of differences (eFigure 18 in Supplement 1). McCrary density tests also found that counties did not bunch on either side of the cutoff or manipulate their quartile classification (eFigure 19 in Supplement 1). Second, our analysis may also have been underpowered to detect statistically significant effects with only 1557 county-observations. But the magnitude of our estimates are consistent with other recent studies examining the effects of benchmark changes. Third, the magnitude of changes in benchmarks at the cutoffs was small compared with the benchmark, ranging from −3.2% to −1.4%. Our estimated associations may not generalize to larger benchmark changes. Further, the associations may be diluted because plans span multiple counties and may face multiple adjustments during a given year. Finally, estimates from the regression discontinuity design are local average treatment effects that apply only to observations that are close to the quartile cutoffs. Estimated associations may not generalize to observations further from the cutoffs.

## Conclusion

In this economic evaluation study, a reduction in the quartile adjustment was associated with small changes in benchmarks, premiums, and the propensity to charge premiums. We found small and nonsignificant changes in other measures of benefit generosity, plan and contract offerings, and MA enrollment rates. This suggests that MA insurers are not sensitive to small changes in payment rates in the short term. This research highlights that modifying the quartile adjustment system may generate savings for CMS without substantially affecting MA beneficiaries.

## Notes

Supplement 1.
eMethods.
**eFigure 1. **Association Between Crossing the Quartile Cutoff and the Quartile Adjustment and Monthly Benchmarks (Average Over 2017-2021) **eFigure 2. **Changes in Monthly Premiums at Quartile Cutoffs (Average Over 2017-2021) **eFigure 3. **Changes in Share of Plans Charging Premiums > $0 at Quartile Cutoffs (Average Over 2017-2021) **eFigure 4. **Changes in Share of Plans using Rebates to Reduce Part B Premiums at Quartile Cutoffs (Average Over 2017-2021) **eFigure 5. **Changes in Average Primary Care Copayments at Quartile Cutoffs (Average Over 2017-2021) **eFigure 6. **Changes in Share of Plans with Supplemental Coverage at Quartile Cutoffs (Average Over 2017-2021) **eFigure 7.** Changes in MA Plans at Quartile Cutoffs (Average Over 2017-2021) **eFigure 8.** Changes in the Number of Contracts at Quartile Cutoffs (Average Over 2017-2021) **eFigure 9.** Changes in Medicare Advantage Enrollment at Quartile Cutoffs (Average Over 2017-2021) **eFigure 10**. First and Second Stage with $20 Bandwidth (Average Over 2017-2021) **eFigure 11. **First and Second Stage with $30 Bandwidth (Average Over 2017-2021) **eFigure 12. **First and Second Stage with $35 Bandwidth (Average Over 2017-2021) **eFigure 13. **First and Second Stage with Quadratic (Average Over 2017-2021) **eFigure 14. **Changes in Outcomes using One-Year Lag **eFigure 15. **Changes in Outcomes using Two-Year Lag **eFigure 16. **Changes in Outcomes using Three-Year Lag **eFigure 17. **Changes in Premiums and Copayments for Plans with Presence in One County, 2017-2021 **eFigure 18. **Changes in Covariates at Quartile Cutoffs (Average Over 2017-2021) **eFigure 19. **McCrary Density Tests at Quartile Cutoffs (Average Over 2017-2021)

Supplement 2. Data sharing statement
