# Supplementary material for: Association Between the Medicare Advantage Quartile Adjustment System and Plan Behavior and Enrollment
Source: JAMA Health Forum. 2024 Jan 12;5(1):e234822. doi: 10.1001/jamahealthforum.2023.4822 (PMC10787313; doi:10.1001/jamahealthforum.2023.4822)
Supplement: Supplement 1. — eMethods. eFigure 1. Association Between Crossing the Quartile Cutoff and the Quartile Adjustment and Monthly Benchmarks (Average Over 2017-2021) eFigure 2. Changes in Monthly Premiums at Quartile Cutoffs (Average Over 2017-2021) eFigure 3. Changes in Share of Plans Charging Premiums > $0 at Quartile Cutoffs (Average Over 2017-2021) eFigure 4. Changes in Share of Plans using Rebates to Reduce Part B Premiums at Quartile Cutoffs (Average Over 2017-2021) eFigure 5. Changes in Average Primary Care Copayments at Quartile Cutoffs (Average Over 2017-2021) eFigure 6. Changes in Share of Plans with Supplemental Coverage at Quartile Cutoffs (Average Over 2017-2021) eFigure 7. Changes in MA Plans at Quartile Cutoffs (Average Over 2017-2021) eFigure 8. Changes in the Number of Contracts at Quartile Cutoffs (Average Over 2017-2021) eFigure 9. Changes in Medicare Advantage Enrollment at Quartile Cutoffs (Average Over 2017-2021) eFigure 10. First and Second Stage with $20 Bandwidth (Average Over 2017-2021) eFigure 11. First and Second Stage with $30 Bandwidth (Average Over 2017-2021) eFigure 12. First and Second Stage with $35 Bandwidth (Average Over 2017-2021) eFigure 13. First and Second Stage with Quadratic (Average Over 2017-2021) eFigure 14. Changes in Outcomes using One-Year Lag eFigure 15. Changes in Outcomes using Two-Year Lag eFigure 16. Changes in Outcomes using Three-Year Lag eFigure 17. Changes in Premiums and Copayments for Plans with Presence in One County, 2017-2021 eFigure 18. Changes in Covariates at Quartile Cutoffs (Average Over 2017-2021) eFigure 19. McCrary Density Tests at Quartile Cutoffs (Average Over 2017-2021) [file jamahealthforum-e234822-s001.pdf]

Supplemental Online Content

Murray RC, Meyers DJ, Fuse Brown EC, Williams TC, Ryan AM. Association between the Medicare Advantage quartile adjustment system and plan behavior and enrollment. *JAMA Health Forum*. 2023;5(1):e234822. doi:10.1001/jamahealthforum.2023.4822

eMethods.

eFigure 1. Association Between Crossing the Quartile Cutoff and the Quartile Adjustment and Monthly Benchmarks (Average Over 2017-2021)

eFigure 2. Changes in Monthly Premiums at Quartile Cutoffs (Average Over 2017-2021)

eFigure 3. Changes in Share of Plans Charging Premiums > $0 at Quartile Cutoffs (Average Over 2017-2021)

eFigure 4. Changes in Share of Plans using Rebates to Reduce Part B Premiums at Quartile Cutoffs (Average Over 2017-2021)

eFigure 5. Changes in Average Primary Care Copayments at Quartile Cutoffs (Average Over 2017-2021)

eFigure 6. Changes in Share of Plans with Supplemental Coverage at Quartile Cutoffs (Average Over 2017-2021)

eFigure 7. Changes in MA Plans at Quartile Cutoffs (Average Over 2017-2021)

eFigure 8. Changes in the Number of Contracts at Quartile Cutoffs (Average Over 2017-2021)

eFigure 9. Changes in Medicare Advantage Enrollment at Quartile Cutoffs (Average Over 2017-2021)

eFigure 10. First and Second Stage with $20 Bandwidth (Average Over 2017-2021)

eFigure 11. First and Second Stage with $30 Bandwidth (Average Over 2017-2021)

eFigure 12. First and Second Stage with $35 Bandwidth (Average Over 2017-2021)

eFigure 13. First and Second Stage with Quadratic (Average Over 2017-2021)

© 2024 Murray RC et al. *JAMA Health Forum.*

eFigure 14. Changes in Outcomes using One-Year Lag

eFigure 15. Changes in Outcomes using Two-Year Lag

eFigure 16. Changes in Outcomes using Three-Year Lag

eFigure 17. Changes in Premiums and Copayments for Plans with Presence in One County, 2017-2021

eFigure 18. Changes in Covariates at Quartile Cutoffs (Average Over 2017-2021)

eFigure 19. McCrary Density Tests at Quartile Cutoffs (Average Over 2017-2021)

This supplemental material has been provided by the authors to give readers additional information about their work.

© 2024 Murray RC et al. *JAMA Health Forum.*

eMethods

Benchmark Setting

Benchmarks are determined by the product of historical Traditional Medicare (TM) spending per capita (i.e., historical TM spending) and the quartile adjustment. To calculate the base amount in each county, Medicare identifies the national per capita Traditional Medicare spending and multiplies it by a county-level geographic index (i.e., the average geographic adjustment, AGA). The AGA is determined using a rolling average of claims data for Traditional Medicare beneficiaries in each county from seven to three years prior and includes weighting for enrollment and risk scores.

To determine the quartile adjustment, counties are ranked based on the prior year historical TM spending and then placed into quartiles. Counties in the lowest spending quartile have their current year historical TM spending adjusted by 115%. Counties in the next quartile receive a 107.5% adjustment. Counties in the third quartile receive a 100% adjustment. And finally, the highest spending counties receive a 95% adjustment.

County-level benchmarks may be different from the combination of the current year historical TM spending and the quartile adjustment if (1) the benchmarks are capped at a level they would have been absent the Affordable Care Act, (2) the quartile designation switches from the previous year, or (3) the county is considered a “qualifying county.” Qualifying counties are those with (1) lower-than-average per capita spending in original Medicare; (2) 25% or more beneficiaries enrolled in MA, as of December 2009; and (3) a payment rate in 2004 based on the minimum amount applicable to a metropolitan statistical area (i.e., an urban floor rate). Further, benchmarks can be adjusted at the plan-level based on plan star rating (i.e., +5 percentage points for 4-5 stars) or if the plan is new or has low enrollment (+3.5 percentage points). After benchmarks are set, plans submit bids relative to the benchmarks. If a plan bids above the benchmark, which is now rare, enrolled beneficiaries must pay the difference between the bid and benchmark in the form of additional premiums. If a plan bids below the benchmark, the plan receives a rebate, which must be returned to beneficiaries via premium support, enhanced benefits, or reduced cost sharing.

Regression Specification

We used a fuzzy regression discontinuity design for all 12 outcomes, which was estimated at the county-level. For the first stage, we estimate the quartile adjustment using the following approach for each county:

© 2024 Murray RC et al. *JAMA Health Forum.*

$$\widehat{QuartileAdjustment} = \alpha_0 + \alpha_1\ TMspend + \delta\ \boldsymbol{Quartile} + \gamma\ \boldsymbol{Cutoff} + \pi\ \boldsymbol{TMspend} \times \boldsymbol{Cutoff} + \sigma\ \boldsymbol{X} + \eta$$

Where $TMspend$ is the historical Traditional Medicare spending from the prior year, used to identify the county's quartile and quartile adjustment factor. $\boldsymbol{Quartile}$ is a vector of dummy variables representing the quartile placement. $\boldsymbol{Cutoff}$ is a vector of dummy variables indicating which cutoff (25th, 50th, or 75th percentile) the county is subject to. And $\boldsymbol{X}$ is a vector of county-level covariates (e.g., average beneficiary age, share of female beneficiaries, unemployment rate).

Then we estimate the second stage for each outcome, Y:

$$Y = \beta_0 + \beta_1\ \widehat{QuartileAdjustment} + \beta_2\ TMspend + \mu\ \boldsymbol{Cutoff} + \rho\ \boldsymbol{TMspend} \times \boldsymbol{Cutoff} + \tau\ \boldsymbol{X} + \xi$$

All regressions are weighted by the county-level Medicare population (i.e., all beneficiaries enrolled in Part A and Part B benefits).

eFigure 1. Association Between Crossing the Quartile Cutoff and the Quartile Adjustment and Monthly Benchmarks (Average Over 2017-2021)

Panel A. First Stage

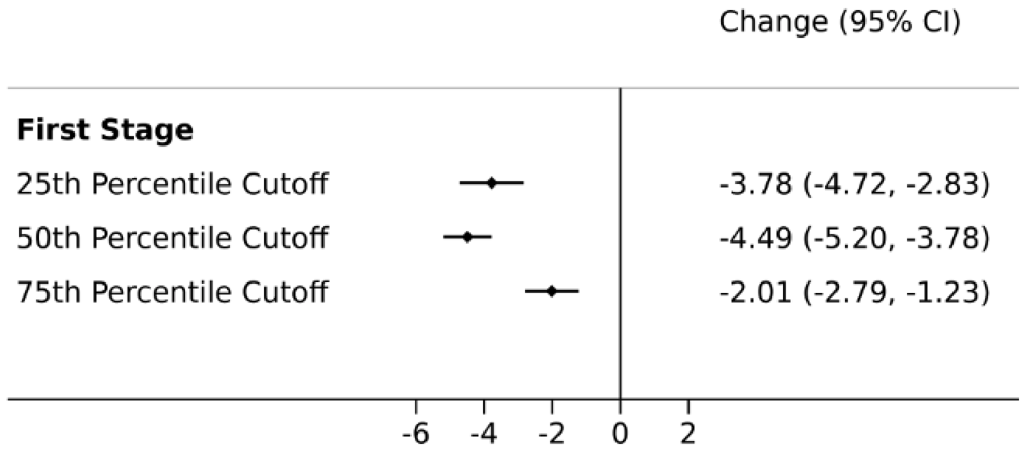

Panel B. Benchmarks

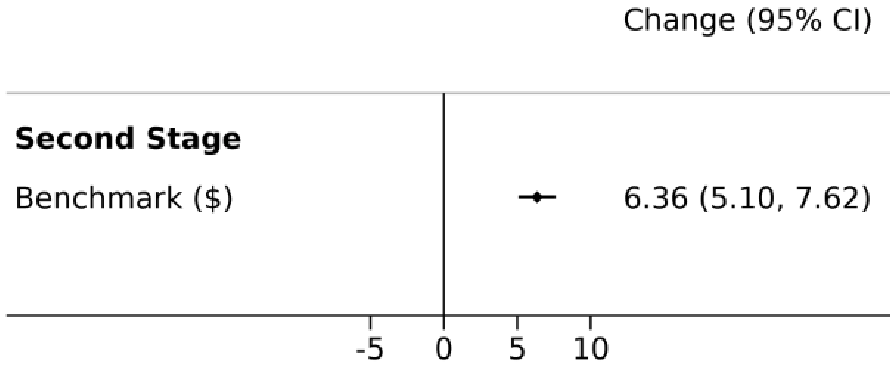

Source: Authors' analysis of 2017-2021 MA Ratebook data.

Notes: MA = Medicare Advantage. TM = Traditional Medicare. CI = Confidence Interval. n = 1,557. The forest plot shows sizable differences in quartile adjustments as counties cross the quartile cutoff. There is a strong association between the quartile adjustment and county-level benchmarks.

© 2024 Murray RC et al. *JAMA Health Forum.*

eFigure 2. Changes in Monthly Premiums at Quartile Cutoffs (Average Over 2017-2021)

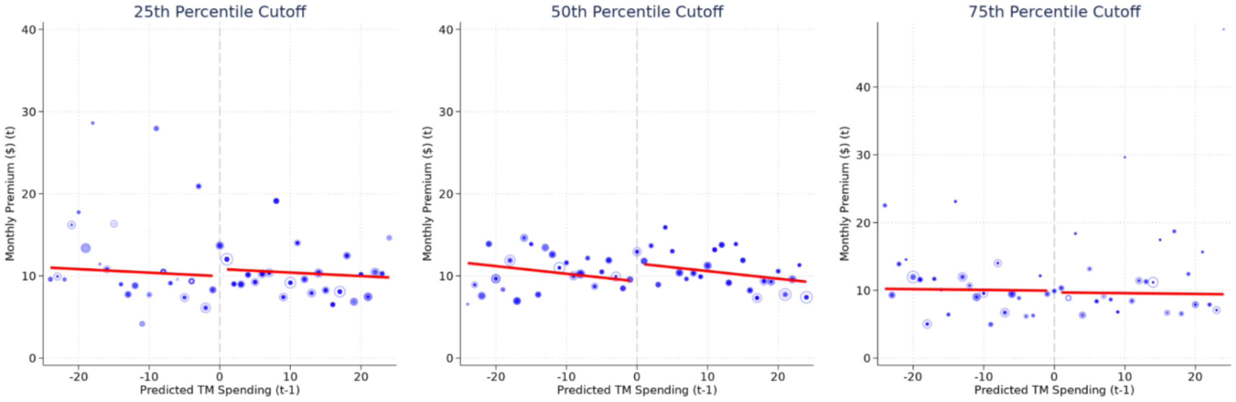

Source: Authors' analysis of 2017-2021 MA Ratebook data.

Notes: MA = Medicare Advantage. TM = Traditional Medicare. The blue dot represents the average for quartile adjustment (t) or benchmark (t) for counties that have a prior year predicted TM spending (t-1) that far above or below the cutoff. The red line represents the fitted relationship between the prior year predicted TM spending (t-1) and monthly premiums (t). The dotted lines represent the quartile cutoffs (t), where counties experience different adjustments to their current year predicted TM spending (t) (i.e., the adjustment) based on quartile placement.

eFigure 3. Changes in Share of Plans Charging Premiums > $0 at Quartile Cutoffs (Average Over 2017-2021)

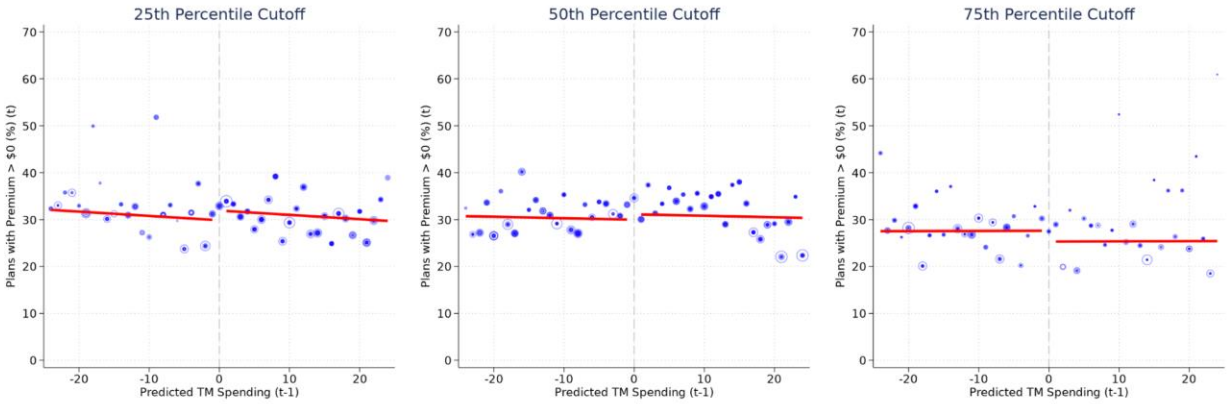

Source: Authors' analysis of 2017-2021 MA Ratebook data.

Notes: MA = Medicare Advantage. TM = Traditional Medicare. The blue dot represents the average for quartile adjustment (t) or benchmark (t) for counties that have a prior year predicted TM spending (t-1) that far above or below the cutoff. The red line represents the fitted relationship between the prior year predicted TM spending (t-1) and the share of plans with >$0 premiums (t). The dotted lines represent the quartile cutoffs (t), where counties experience different adjustments to their current year predicted TM spending (t) (i.e., the adjustment) based on quartile placement.

© 2024 Murray RC et al. *JAMA Health Forum.*

eFigure 4. Changes in Share of Plans using Rebates to Reduce Part B Premiums at Quartile Cutoffs (Average Over 2017-2021)

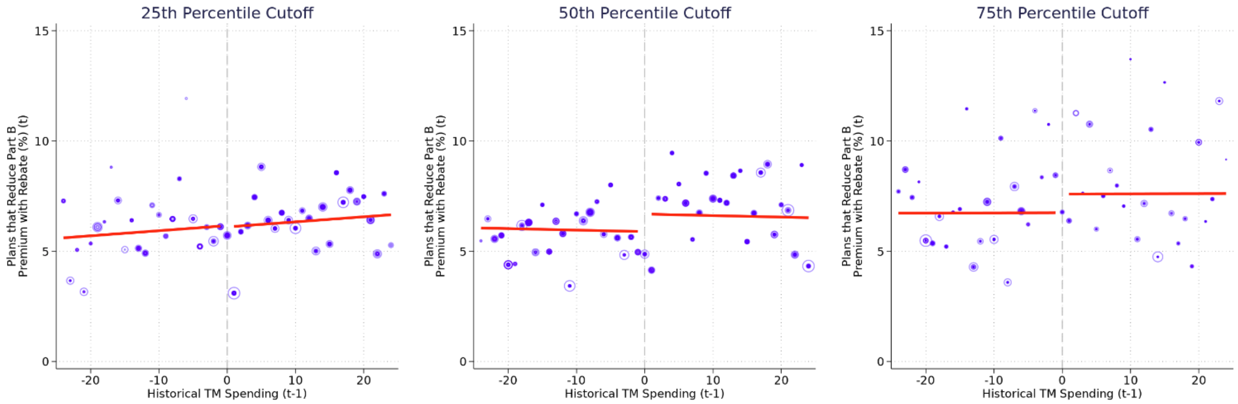

Source: Authors' analysis of 2017-2021 MA Ratebook data.

Notes: MA = Medicare Advantage. TM = Traditional Medicare. The blue dot represents the average for quartile adjustment (t) or benchmark (t) for counties that have a prior year predicted TM spending (t-1) that far above or below the cutoff. The red line represents the fitted relationship between the prior year predicted TM spending (t-1) and the share of plans using rebates to reduce Part B premiums (t). The dotted lines represent the quartile cutoffs (t), where counties experience different adjustments to their current year predicted TM spending (t) (i.e., the adjustment) based on quartile placement.

eFigure 5. Changes in Average Primary Care Copayments at Quartile Cutoffs (Average Over 2017-2021)

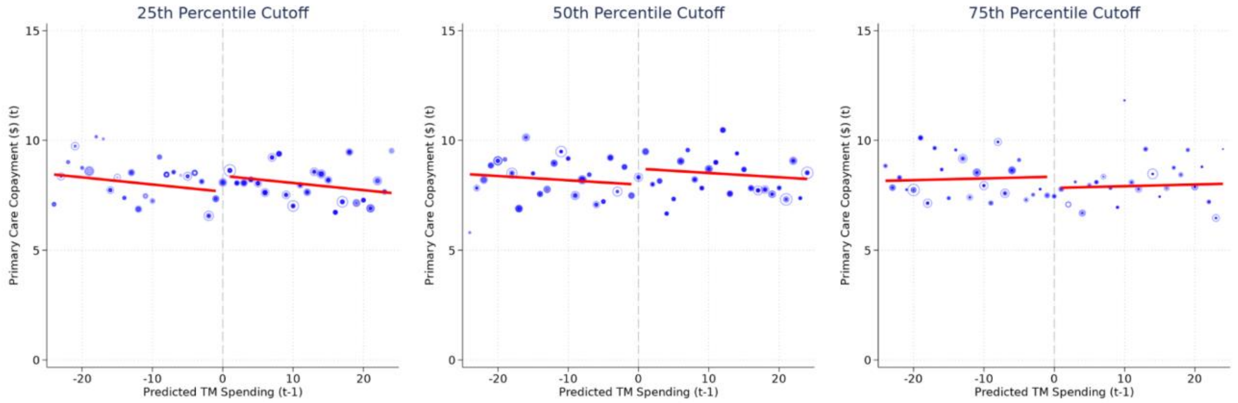

Source: Authors' analysis of 2017-2021 MA Ratebook data.

Notes: MA = Medicare Advantage. TM = Traditional Medicare. The blue dot represents the average for quartile adjustment (t) or benchmark (t) for counties that have a prior year predicted TM spending (t-1) that far above or below the cutoff. The red line represents the fitted relationship between the prior year predicted TM spending (t-1) and primary care copays (t). The dotted lines represent the quartile cutoffs (t), where counties experience different adjustments to their current year predicted TM spending (t) (i.e., the adjustment) based on quartile placement.

eFigure 6. Changes in Share of Plans with Supplemental Coverage at Quartile Cutoffs (Average Over 2017-2021)

© 2024 Murray RC et al. *JAMA Health Forum.*

Panel A. Preventive Dental Coverage

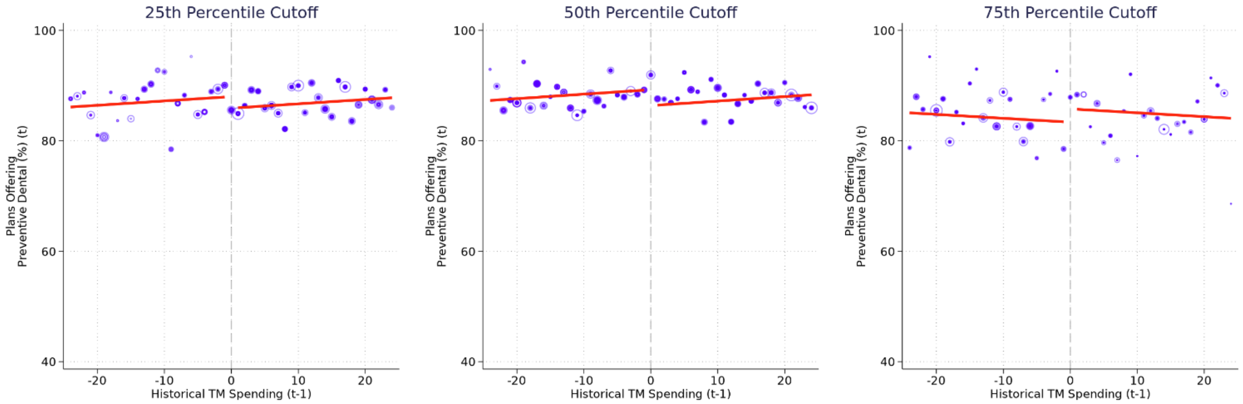

Panel B. Comprehensive Dental Coverage

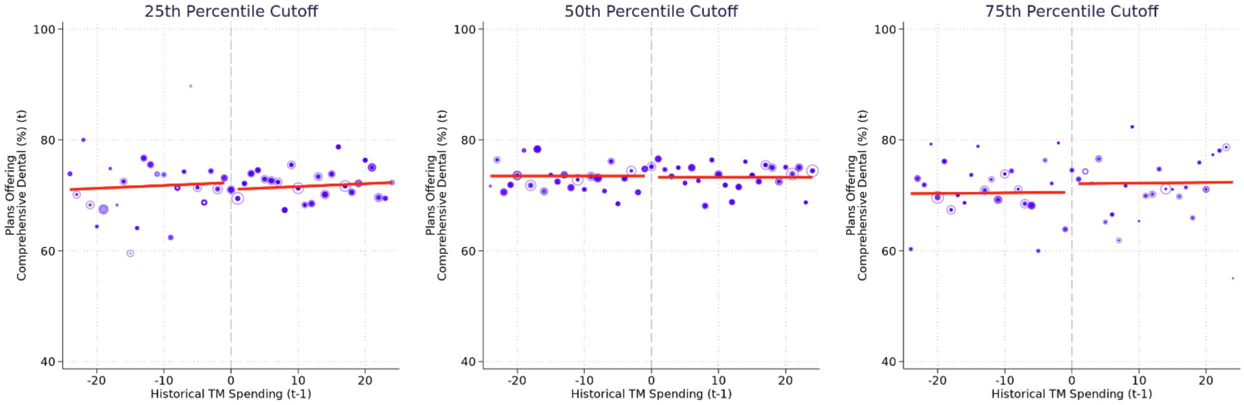

Panel C. Eye Exam Coverage

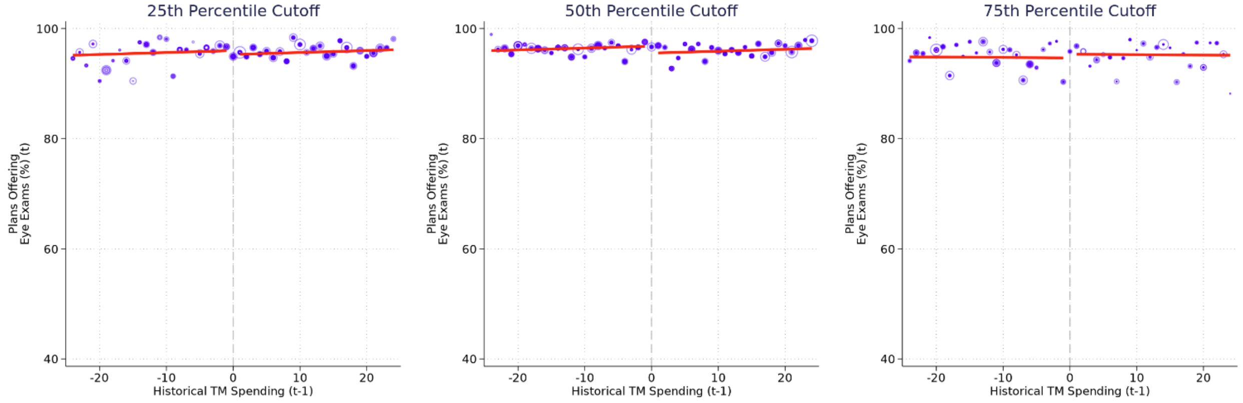

Panel D. Eyewear Coverage

© 2024 Murray RC et al. *JAMA Health Forum.*

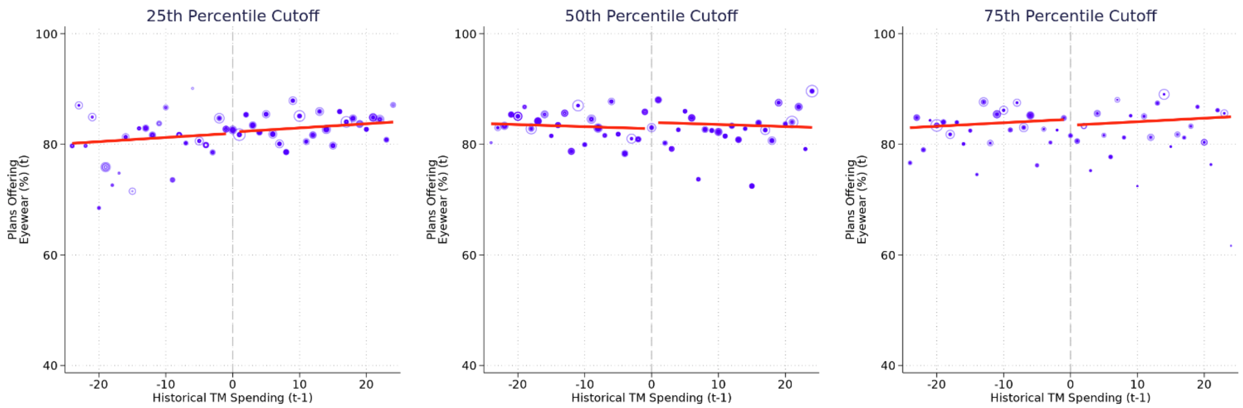

Panel E. Hearing Exam Coverage

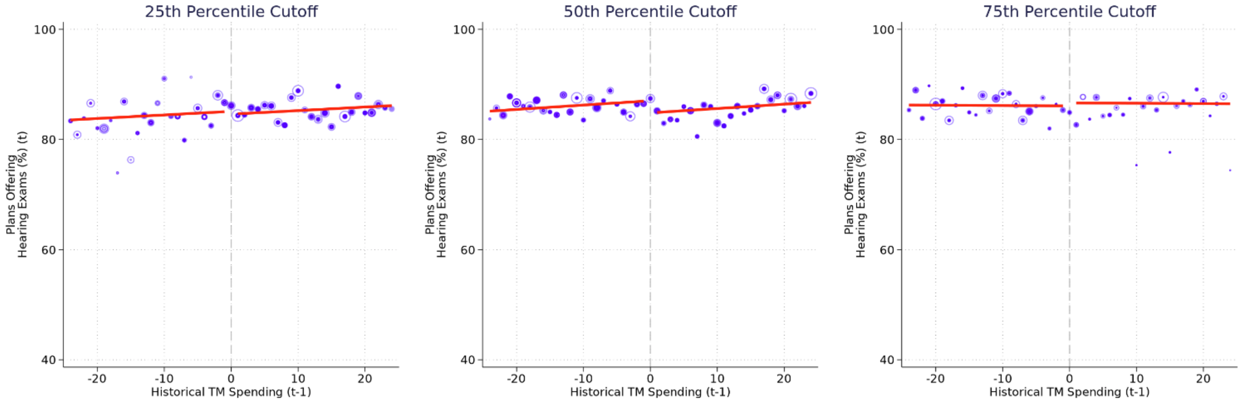

Panel F. Hearing Aid Coverage

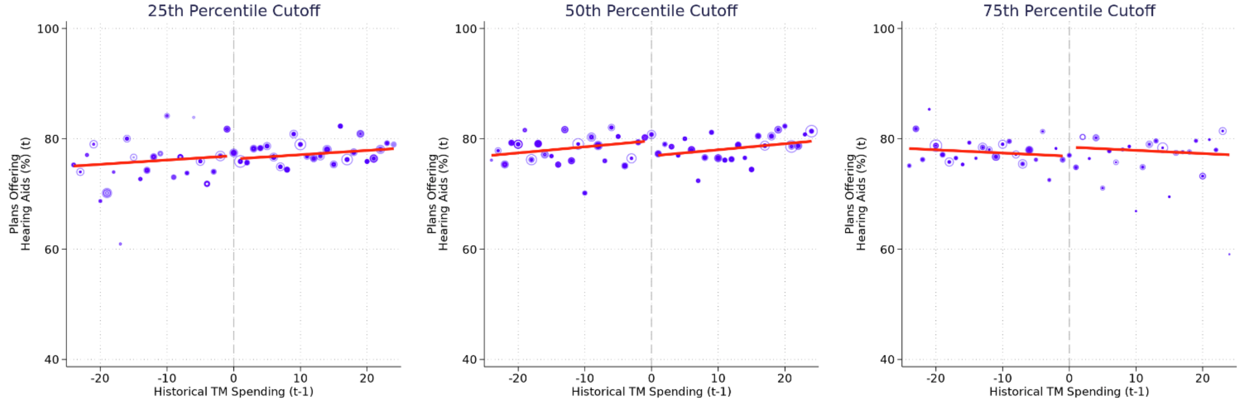

Source: Authors' analysis of 2017-2021 MA Ratebook data.

Notes: MA = Medicare Advantage. TM = Traditional Medicare. The blue dot represents the average for quartile adjustment (t) or benchmark (t) for counties that have a prior year predicted TM spending (t-1) that far above or below the cutoff. The red line represents the fitted relationship between the prior year predicted TM spending (t-1) and share of supplemental benefits (t). The dotted lines represent the quartile cutoffs (t), where counties experience different adjustments to their current year predicted TM spending (t) (i.e., the adjustment) based on quartile placement.

© 2024 Murray RC et al. *JAMA Health Forum.*

eFigure 7. Changes in MA Plans at Quartile Cutoffs (Average Over 2017-2021)

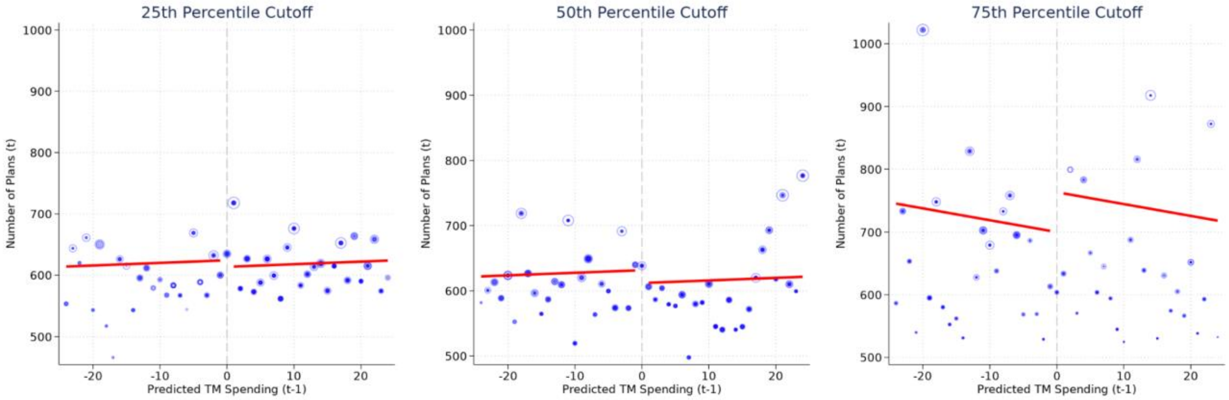

Source: Authors' analysis of 2017-2021 MA Ratebook data.

Notes: MA = Medicare Advantage. TM = Traditional Medicare. The blue dot represents the average for quartile adjustment (t) or benchmark (t) for counties that have a prior year predicted TM spending (t-1) that far above or below the cutoff. The red line represents the fitted relationship between the prior year predicted TM spending (t-1) and MA plans (t). The dotted lines represent the quartile cutoffs (t), where counties experience different adjustments to their current year predicted TM spending (t) (i.e., the adjustment) based on quartile placement.

eFigure 8. Changes in the Number of Contracts at Quartile Cutoffs (Average Over 2017-2021)

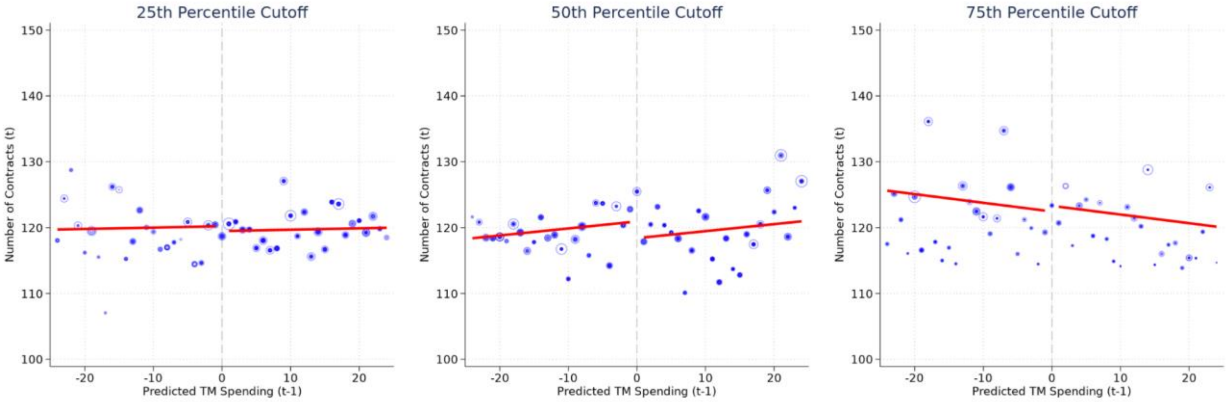

Source: Authors' analysis of 2017-2021 MA Ratebook data.

Notes: MA = Medicare Advantage. TM = Traditional Medicare. The blue dot represents the average for quartile adjustment (t) or benchmark (t) for counties that have a prior year predicted TM spending (t-1) that far above or below the cutoff. The red line represents the fitted relationship between the prior year predicted TM spending (t-1) and MA contracts (t). The dotted lines represent the quartile cutoffs (t), where counties experience different adjustments to their current year predicted TM spending (t) (i.e., the adjustment) based on quartile placement.

eFigure 9. Changes in Medicare Advantage Enrollment at Quartile Cutoffs (Average Over 2017-2021)

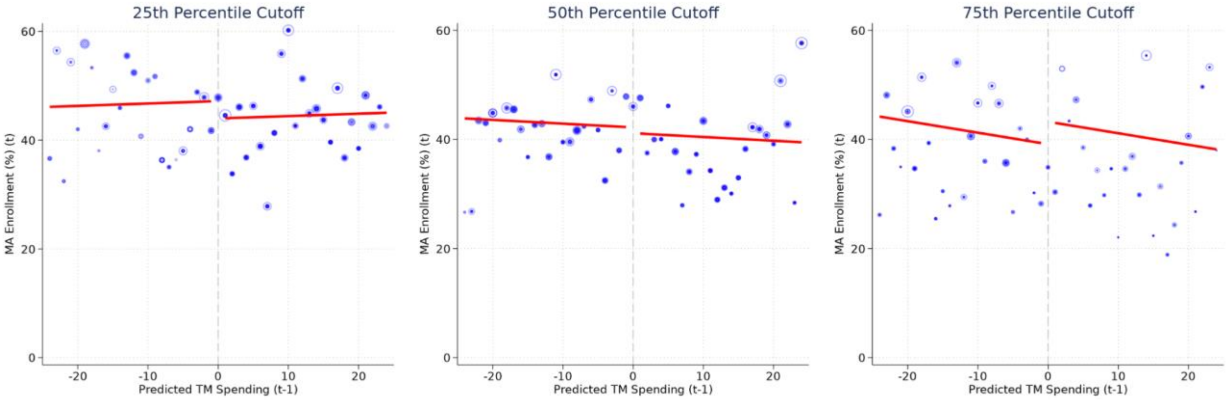

Source: Authors' analysis of 2017-2021 MA Ratebook data.

Notes: MA = Medicare Advantage. TM = Traditional Medicare. The blue dot represents the average for quartile adjustment (t) or benchmark (t) for counties that have a prior year predicted TM spending (t-1) that far above or below the cutoff. The red line represents the fitted relationship between the prior year predicted TM spending (t-1) and MA enrollment (t). The dotted lines represent the quartile cutoffs (t), where counties experience different adjustments to their current year predicted TM spending (t) (i.e., the adjustment) based on quartile placement.

© 2024 Murray RC et al. *JAMA Health Forum.*

eFigure 10. First and Second Stage with $20 Bandwidth (Average Over 2017-2021)

Panel A. First Stage

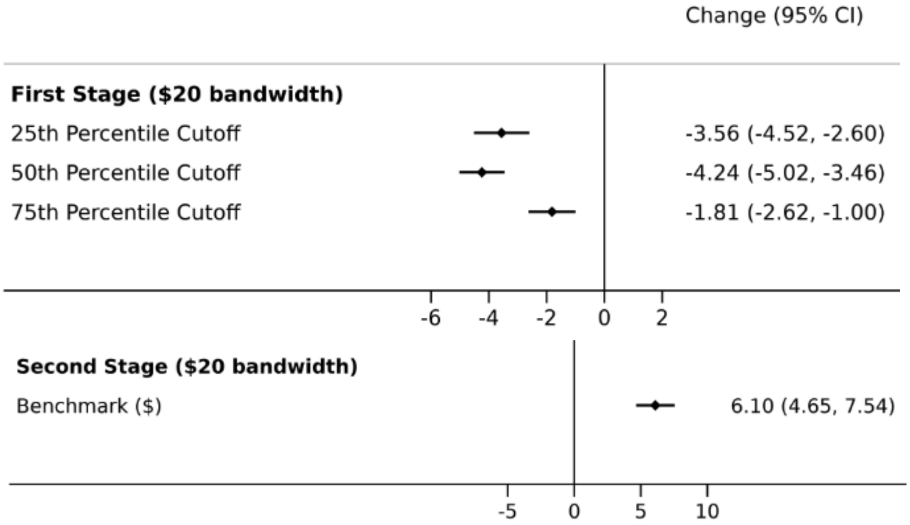

Panel B. Second Stage

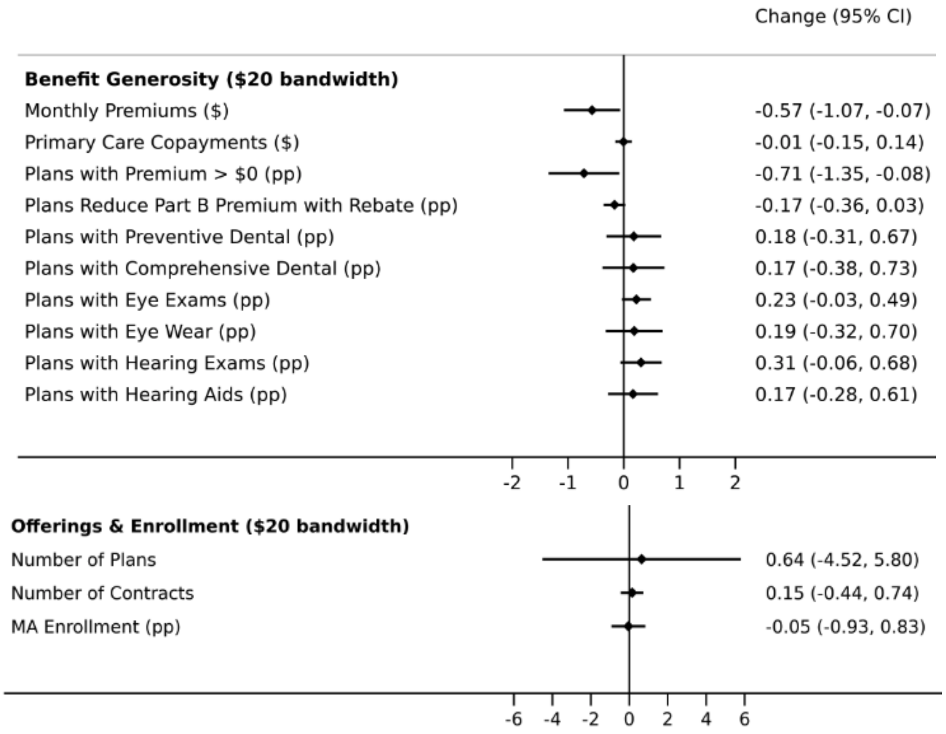

Source: Authors' analysis of 2017-2021 MA Ratebook data.

Notes: MA = Medicare Advantage. Observations = 1,304.

© 2024 Murray RC et al. *JAMA Health Forum.*

eFigure 11. First and Second Stage with $30 Bandwidth (Average Over 2017-2021)

Panel A. First Stage

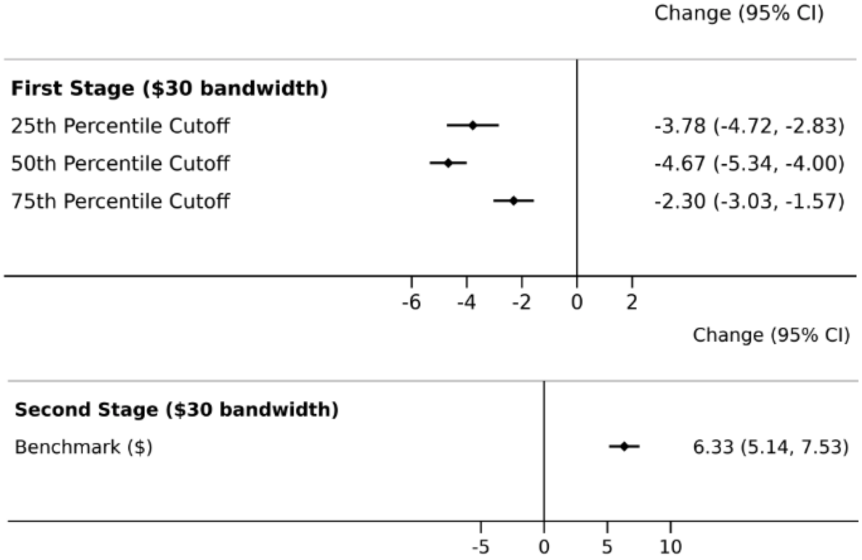

Panel B. Second Stage

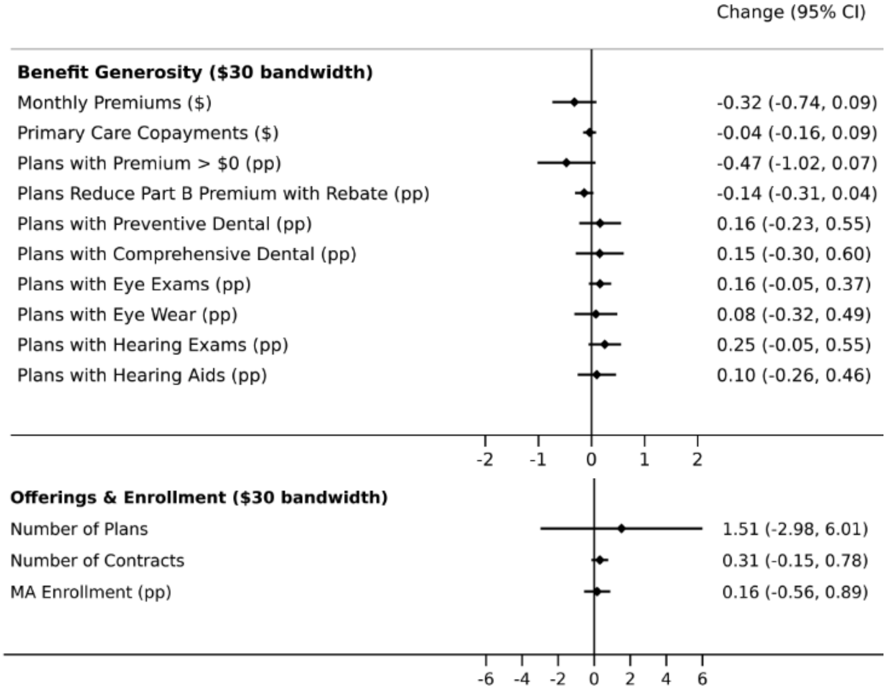

Source: Authors' analysis of 2017-2021 MA Ratebook data.

Notes: MA = Medicare Advantage. Observations = 1,700.

© 2024 Murray RC et al. *JAMA Health Forum.*

eFigure 12. First and Second Stage with $35 Bandwidth (Average Over 2017-2021)

Panel A. First Stage

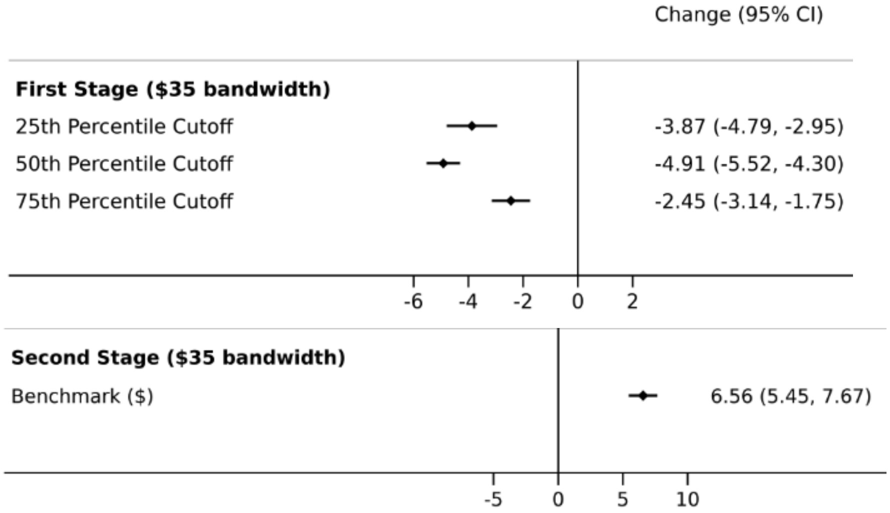

Panel B. Second Stage

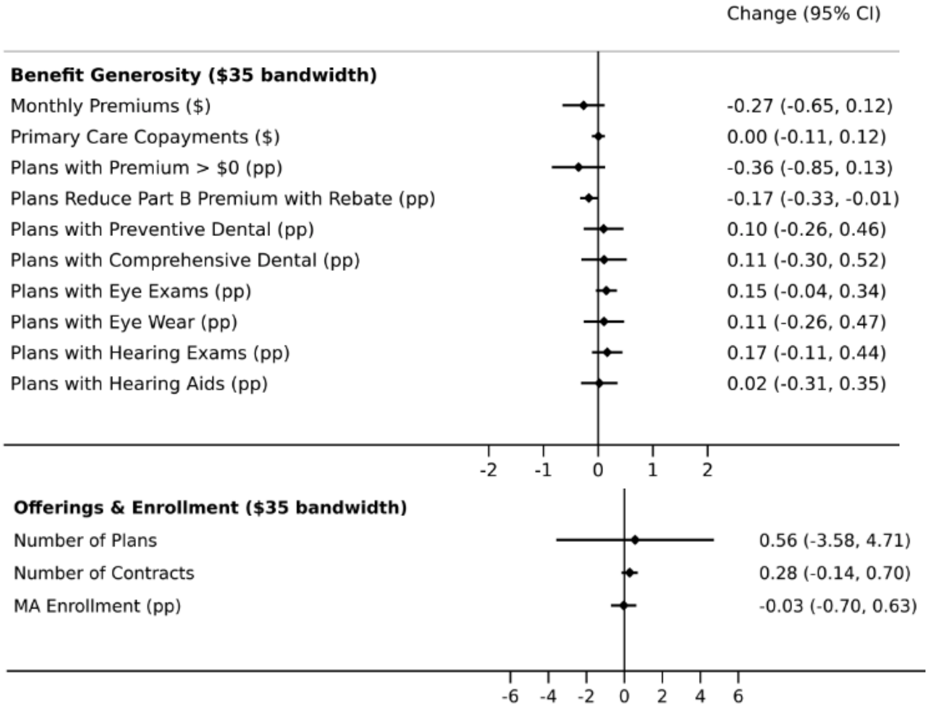

Source: Authors' analysis of 2017-2021 MA Ratebook data.

Notes: MA = Medicare Advantage. Observations = 1,890.

© 2024 Murray RC et al. *JAMA Health Forum.*

eFigure 13. First and Second Stage with Quadratic (Average Over 2017-2021)

Panel A. First Stage

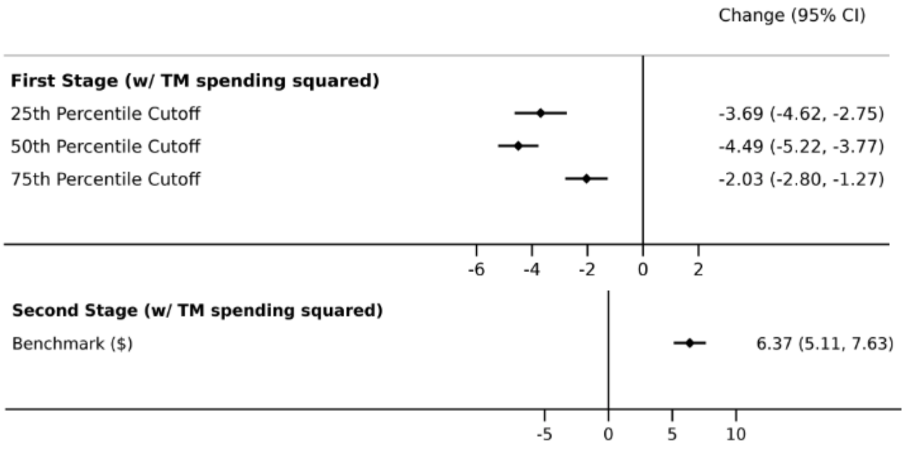

Panel B. Second Stage

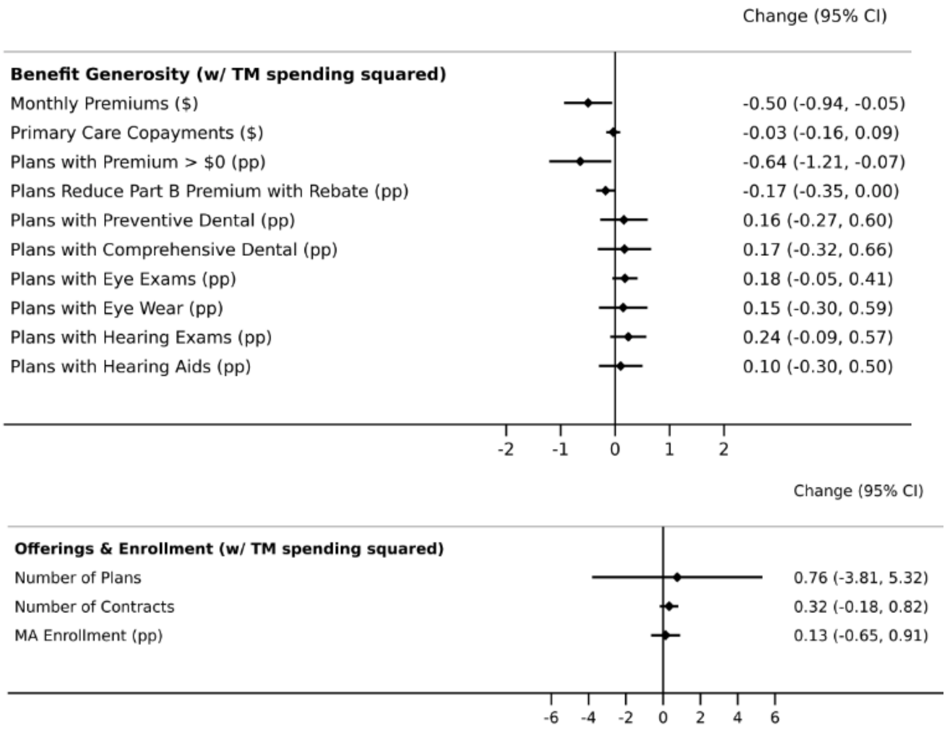

Source: Authors' analysis of 2017-2021 MA Ratebook data.

Notes: MA = Medicare Advantage. Observations = 1,557.

© 2024 Murray RC et al. *JAMA Health Forum.*

eFigure 14. Changes in Outcomes using One-Year Lag

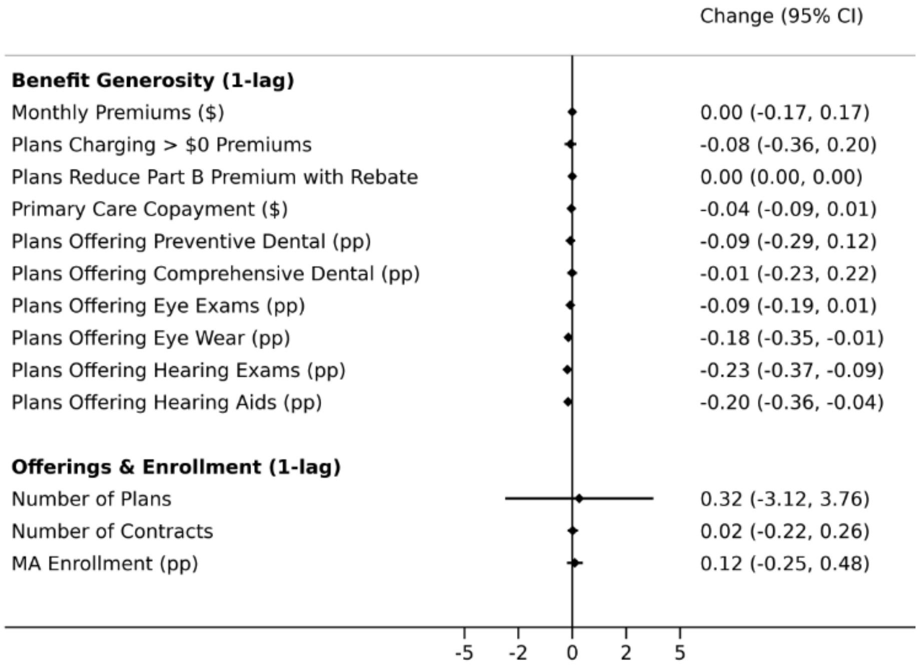

Source: Authors' analysis of 2017-2021 MA Ratebook data.

Notes: MA = Medicare Advantage. TM = Traditional Medicare.

eFigure 15. Changes in Outcomes using Two-Year Lag

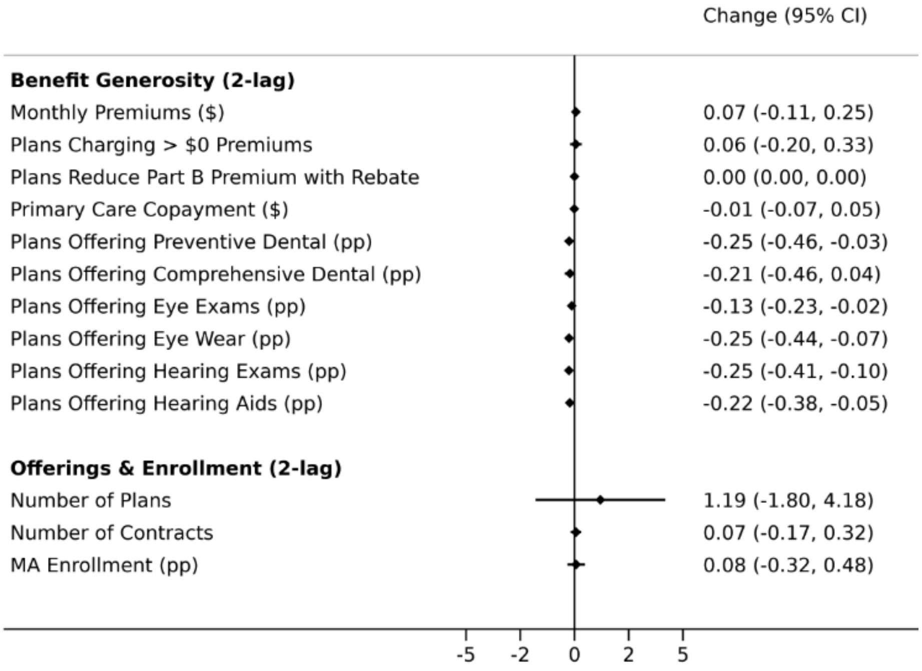

Source: Authors' analysis of 2017-2021 MA Ratebook data.

Notes: MA = Medicare Advantage.

© 2024 Murray RC et al. *JAMA Health Forum.*

eFigure 16. Changes in Outcomes using Three-Year Lag

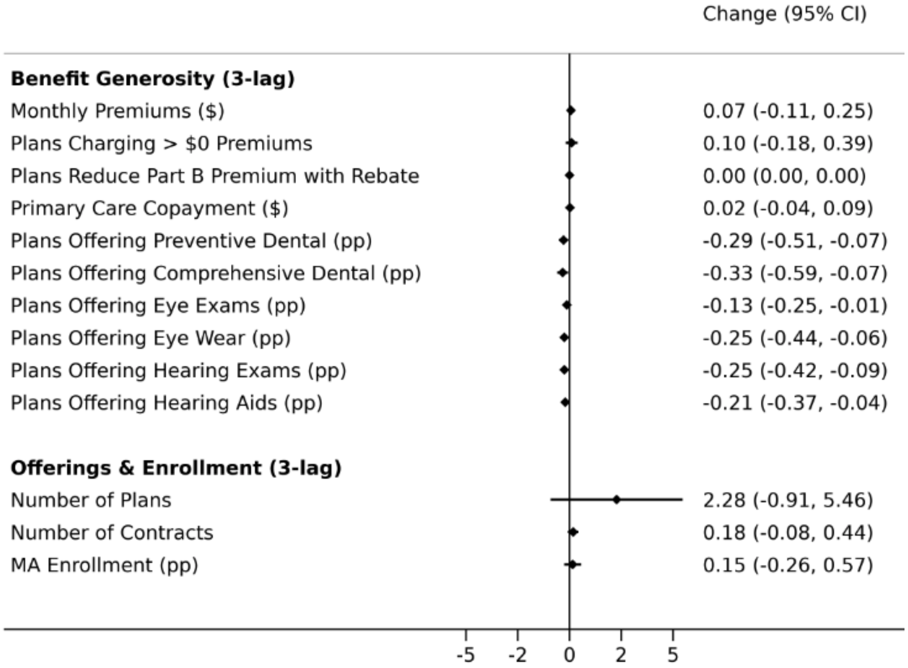

Source: Authors' analysis of 2017-2021 MA Ratebook data.

Notes: MA = Medicare Advantage.

eFigure 17. Changes in Premiums and Copayments for Plans with Presence in One County, 2017-2021

Panel A. First Stage

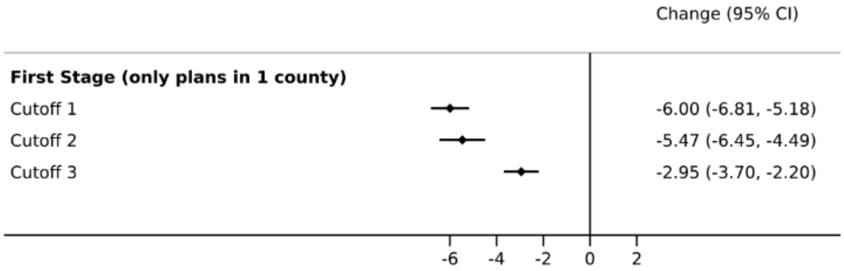

Panel B. Second Stage

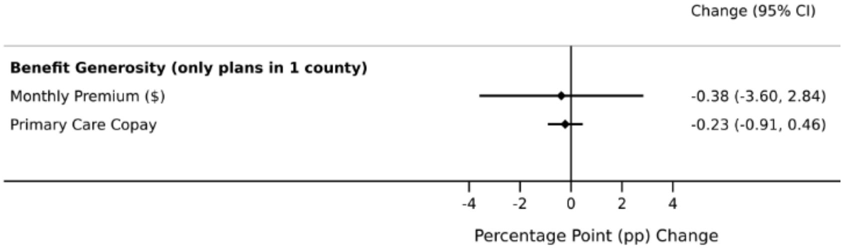

Source: Authors' analysis of 2017-2021 MA Ratebook data.

Notes: MA = Medicare Advantage. Observations = 175

eFigure 18. Changes in Covariates at Quartile Cutoffs (Average Over 2017-2021)

© 2024 Murray RC et al. *JAMA Health Forum.*

Panel A. Average Beneficiary Age

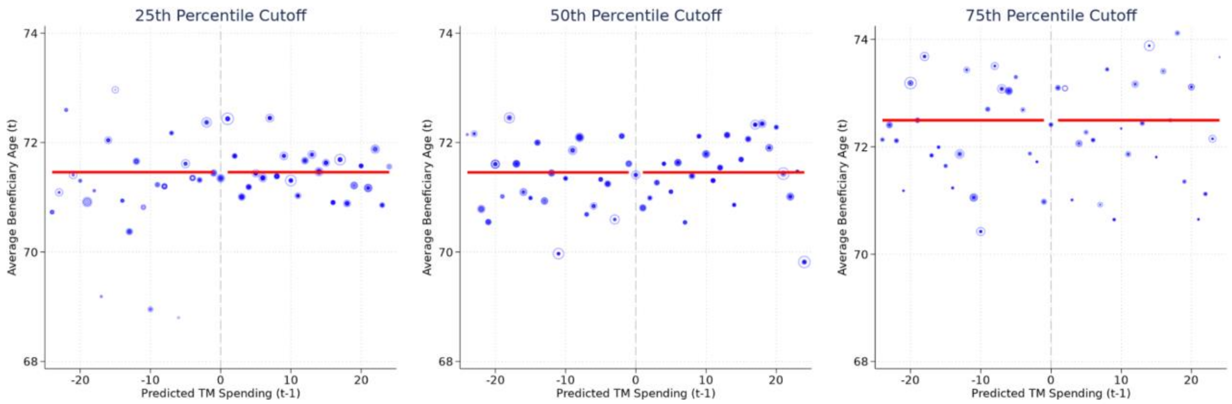

Panel B. Share Female

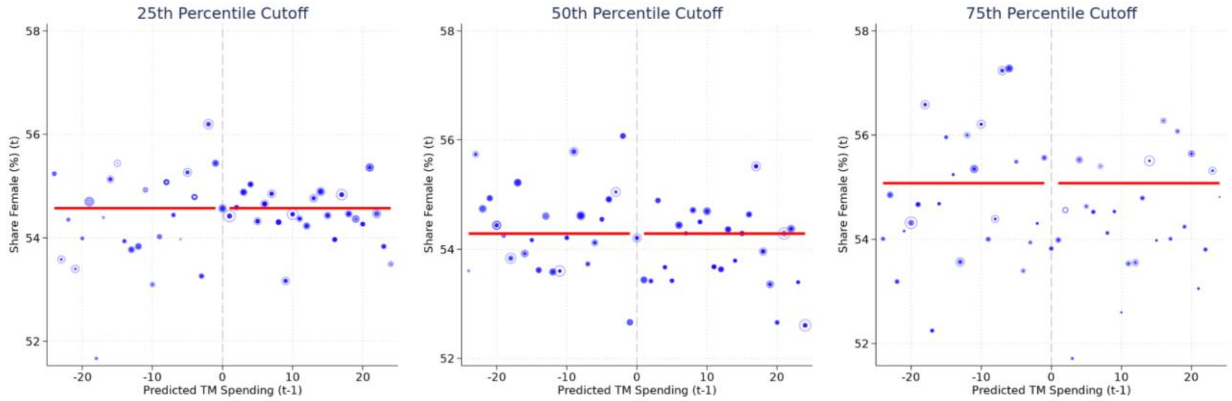

Panel C. Unemployment Rate

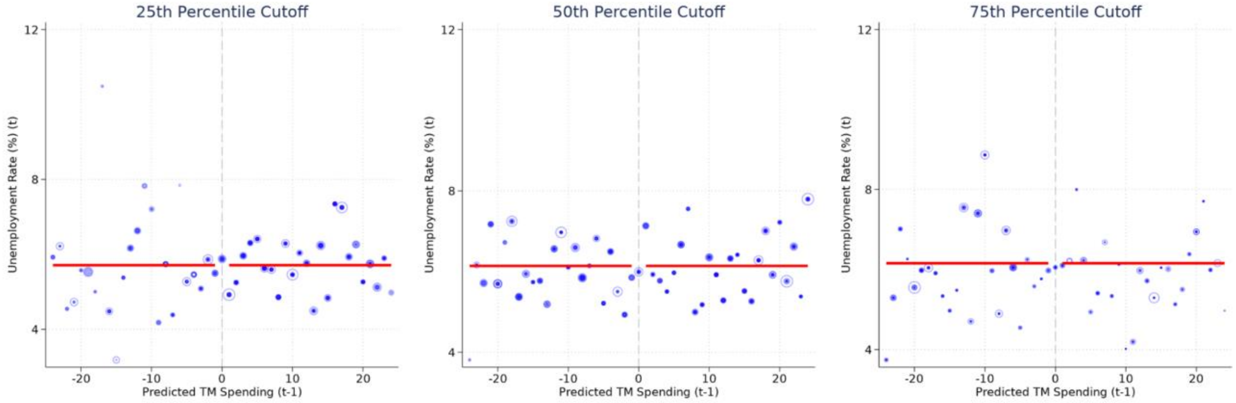

Panel D. Income Per Capita (in Thousands)

© 2024 Murray RC et al. *JAMA Health Forum.*

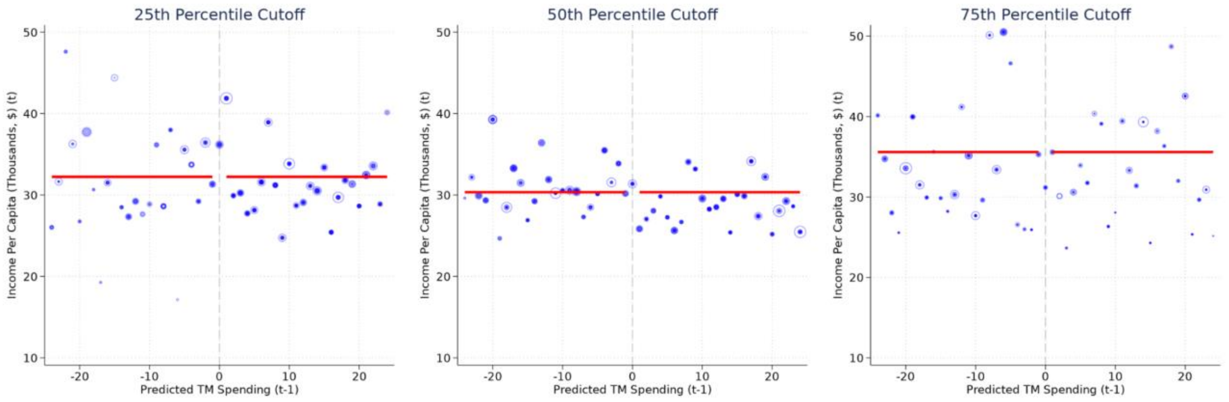

Panel E. Average Beneficiary Risk Score

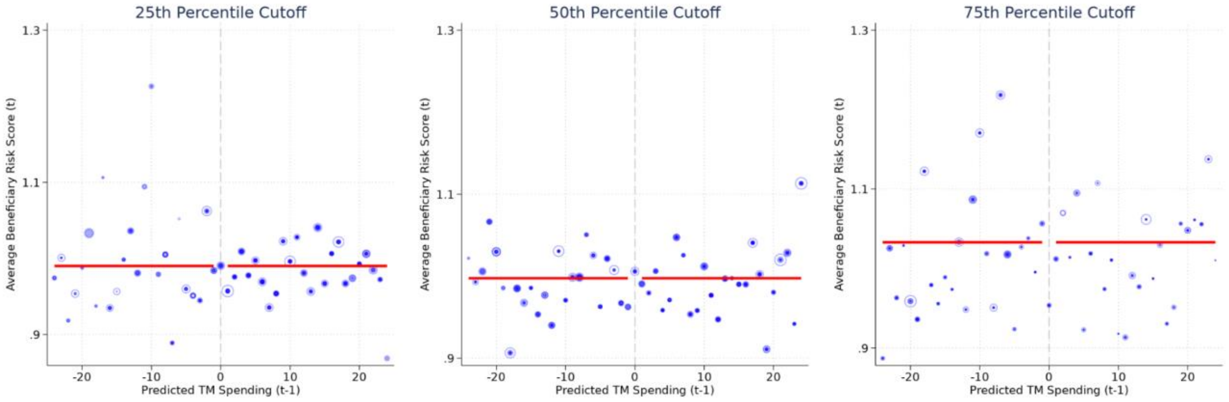

Panel F. Share in Urban Areas

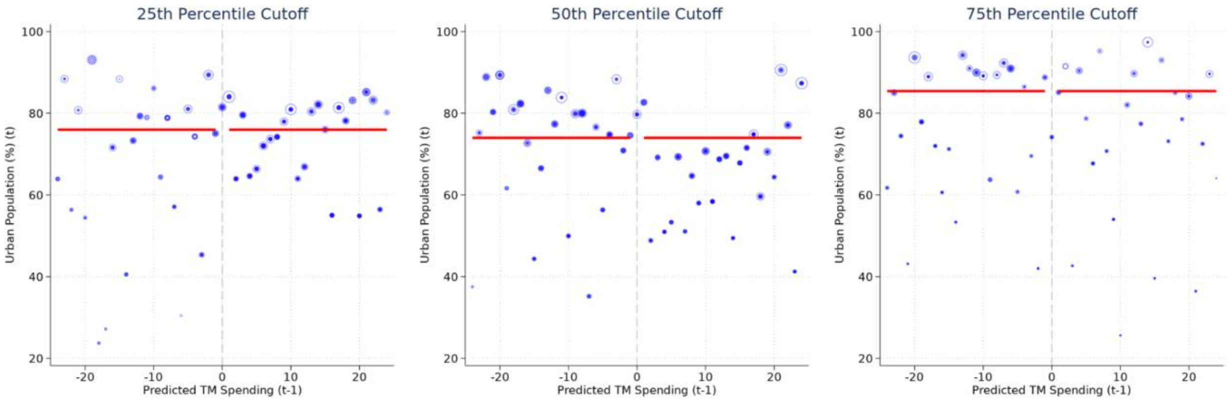

Panel G. County Population (in Millions)

© 2024 Murray RC et al. *JAMA Health Forum.*

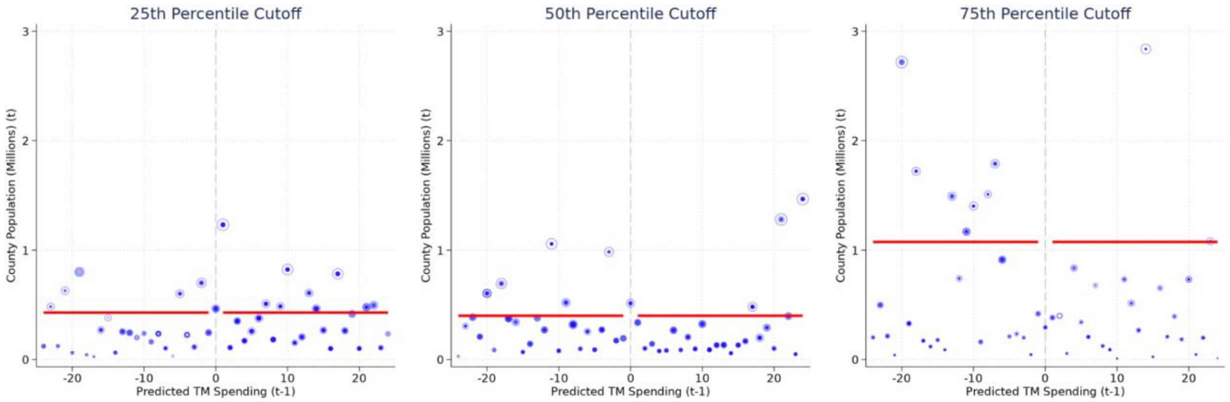

Source: Authors' analysis of 2017-2021 MA Ratebook data.

Notes: MA = Medicare Advantage. TM = Traditional Medicare. The blue dot represents the average for quartile adjustment (t) or benchmark (t) for counties that have a prior year predicted TM spending (t-1) that far above or below the cutoff. The red line represents the fitted relationship between the prior year predicted TM spending (t-1) and the covariate (t). The dotted lines represent the quartile cutoffs (t), where counties experience different adjustments to their current year predicted TM spending (t) (i.e., the adjustment) based on quartile placement.

eFigure 19. McCrary Density Tests at Quartile Cutoffs (Average Over 2017-2021)

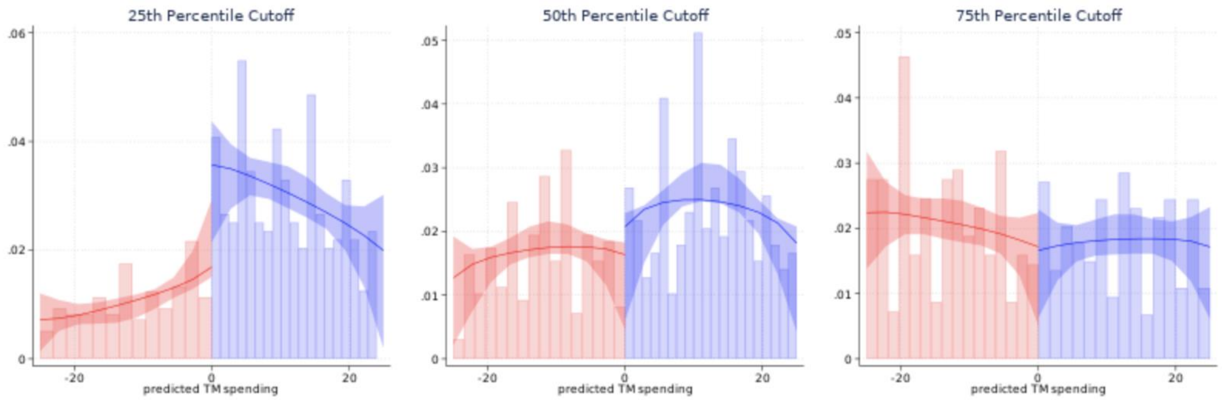

Source: Authors' analysis of 2017-2021 MA Ratebook data.

Notes: TM = Traditional Medicare.

© 2024 Murray RC et al. *JAMA Health Forum.*
