# Supplementary material for: Association Between the Medicare Advantage Quartile Adjustment System and Plan Behavior and Enrollment
Source: JAMA Health Forum. 2024 Jan 12;5(1):e234822. doi: 10.1001/jamahealthforum.2023.4822 (PMC10787313; doi:10.1001/jamahealthforum.2023.4822)
Supplement: Supplement 2. — Data sharing statement [file jamahealthforum-e234822-s002.pdf]

Data Sharing Statement

Murray. Association Between the Medicare Advantage Quartile Adjustment System and Plan Behavior and Enrollment. *JAMA Health Forum*. Published January 12, 2024.
doi:10.1001/jamahealthforum.2023.4822

Data
Data available: No
